# BELHD: improving biomedical entity linking with homonym disambiguation

**DOI:** 10.1093/bioinformatics/btae474

**Published:** 2024-07-27

**Authors:** Samuele Garda, Ulf Leser

**Affiliations:** Computer Science, Humboldt-Universität zu Berlin, Berlin 12489, Germany; Computer Science, Humboldt-Universität zu Berlin, Berlin 12489, Germany

## Abstract

**Motivation:**

Biomedical entity linking (BEL) is the task of grounding entity mentions to a given knowledge base (KB). Recently, neural name-based methods, system identifying the most appropriate name in the KB for a given mention using neural network (either via dense retrieval or autoregressive modeling), achieved remarkable results for the task, without requiring manual tuning or definition of domain/entity-specific rules. However, as name-based methods directly return KB names, they cannot cope with homonyms, i.e. different KB entities sharing the exact same name. This significantly affects their performance for KBs where homonyms account for a large amount of entity mentions (e.g. UMLS and NCBI Gene).

**Results:**

We present BELHD (Biomedical Entity Linking with Homonym Disambiguation), a new name-based method that copes with this challenge. BELHD builds upon the BioSyn model with two crucial extensions. First, it performs pre-processing of the KB, during which it expands homonyms with a specifically constructed disambiguating string, thus enforcing unique linking decisions. Second, it introduces candidate sharing, a novel strategy that strengthens the overall training signal by including similar mentions from the same document as positive or negative examples, according to their corresponding KB identifier. Experiments with 10 corpora and 5 entity types show that BELHD improves upon current neural state-of-the-art approaches, achieving the best results in 6 out of 10 corpora with an average improvement of 4.55pp recall@1. Furthermore, the KB preprocessing is orthogonal to the prediction model and thus can also improve other neural methods, which we exemplify for GenBioEL, a generative name-based BEL approach.

**Availability and implementation:**

The code to reproduce our experiments can be found at: https://github.com/sg-wbi/belhd.

## 1 Introduction

Biomedical entity linking (BEL) (or biomedical named entity normalization) is the task of grounding entity mentions in a text to a Knowledge Base (KB). While early methods in BEL were mostly based on dictionary matching and hand-crafted, entity-specific rule sets (French and McInnes 2022), recent studies show that methods based on neural network, specifically pre-trained language models, for many types of entities can achieve state-of-the-art results without these extra efforts (Sung *et al.* 2020). Approaches in this category can be divided into two main groups [We focus on candidate generation, i.e. we do not consider reranking methods like the cross-encoder; Logeswaran *et al.* 2019, which require a pre-computed candidate list (see Section 4)]. Entity-based methods construct entity representations in form of embeddings followed by a matching step based on dense retrieval (Varma *et al.* 2021, Zhang *et al.* 2022, Agarwal *et al.* 2022 inter alia). In contrast, name-based methods directly identify the best matching name in the KB, either via dense retrieval or using autoregressive modeling (Yuan *et al.* 2022).

**Figure 1.**
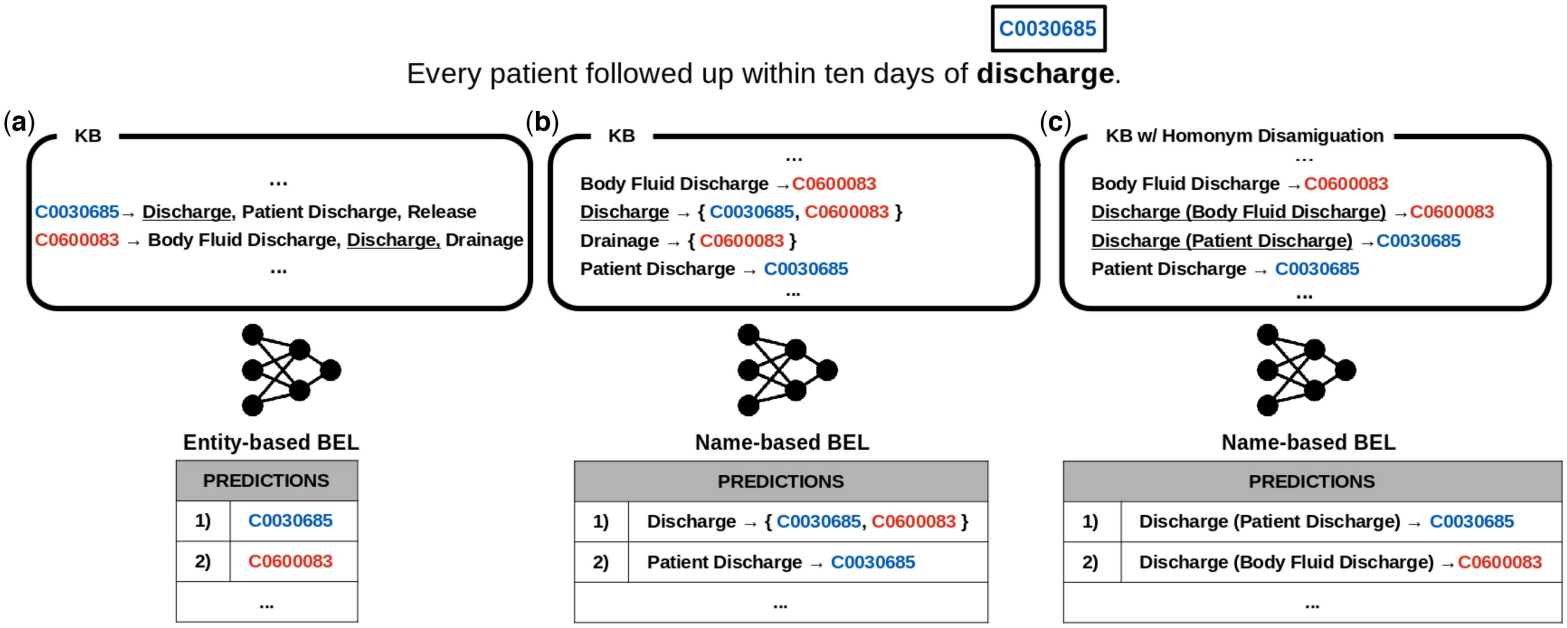
Illustration of the entity-based (a) and name-based (b) approach for biomedical entity linking with pre-trained language models. Underlined text highlights the KB homonym (see Section 2.1) preventing a unique linking decision (b). In (c) we show how in BELHD we address the issue by replacing homonyms with their disambiguated version. Text in blue and red represent the correct and wrong prediction, respectively.

Though name-based methods have been widely investigated and often outperform other approaches in evaluations (Sung *et al.* 2020, Liu *et al.* 2021), they suffer from a flaw that is particularly critical in biomedical applications, i.e. the handling of homonyms. A homonym is a name in a KB that appears more than once, resulting from multiple KB entities having the same name. As shown in Fig. 1, name-based methods are not capable of resolving such cases because they can only return the (ambiguous) name as result (Zhang *et al.* 2022). To circumvent this issue, previous evaluations allow models to return multiple KB entities which are then counted as partly correct (Sung *et al.* 2020). However, in applications BEL is just one step in a complex pipeline, where downstream components typically cannot properly handle non-unique linking information (Wang *et al.* 2022). This means that homonyms severely limit the use of name-based systems for biomedical applications with homonym-rich KBs. This is especially problematic for genes, where homonyms are a key characteristic (Wei and Kao 2011) as the same gene can be found in multiple species, e.g. human versus mouse “*α*2microglobulin” (see Section 2.1.1). For instance, we find that in the NLM-Gene corpus (Islamaj *et al.* 2021) homonyms account for up to 66.1% of the mentions (see Supplementary Data A). A second class of entity mentions that suffers from a large fraction of homonyms are abbreviations, e.g. “TS” either meaning “Tourette Syndrome” or “Timothy Syndrome.” State-of-the-art approaches often try to address such problems using specialized tools, such as Ab3P (Sohn *et al.* 2008) to expand abbreviations or SpeciesAssignment (Luo *et al.* 2022b) to determine the species of gene mentions. These however are non-adaptable solutions crafted for a specific category of homonyms, leaving many other cases unhandled, as the one in the example in Fig. 1.

We introduce BELHD (Biomedical Entity Linking with Homonym Disambiguation), which extends the name-based method BioSyn (Sung *et al.* 2020) to properly handle homonyms. BELHD is based on two novel key ideas. First, it uses a pre-processing step during which it *disambiguates* the respective KB by expanding any instance of a homonym with a disambiguating string. An example is shown in Fig. 1c, in which “Discharge” is a homonym referring both to entity C0030685 and C0600083 in UMLS. We replace both instances with properly expanded names, i.e. “Discharge (Patient Discharge)” and “Discharge (Body Fluid Discharge),” thus enforcing unique linking decision.

Secondly, similar to De Cao *et al.* (2021), BELHD processes the entire text with *all* of its mentions at once (see Supplementary Data G for a visualization). This allows us to introduce *candidate sharing*, a novel strategy to select training candidates in which mentions not only use their own candidates, but also those retrieved for other mentions appearing in the same document. As co-occurring mentions are often related (Chen *et al.* 2021), sharing candidates among mentions pushes the model towards learning patterns that differentiate semantically related concepts having similar contextual features, thus improving the overall training signal (see Section 2.2 for details).

We performed extensive experiments to compare BELHD to SOTA neural entity linking methods, including the two entity-based models arboEL (Agarwal *et al.* 2022) and KRISSBERT (Zhang *et al.* 2022) and the two name-based models BioSyn (Sung *et al.* 2020) and GenBioEL (Yuan *et al.* 2022). Our evaluation involves 10 corpora linked to 6 KBs. We find that BELHD outperforms its neural competitors in 6 of the 10 corpora with an average improvement of 4.55pp recall@1. Additionally, as our approach for homonym disambiguation (HD) is independent of the core model it can also be applied to improve other name-based methods, which we show for GenBioEL.

Compared to domain-specific methods, in particular PubTator3 (Wei *et al.* 2024) and its collection of manually-tuned entity-specific models, BELHD gives better performance for all entity types but genes, for which PubTator3 achieves a significantly higher precision (see Section 4), probably because of its extensive usage of manually curated entity dictionaries. Our results suggest that neural methods, and BELHD in particular, can be a valid all-in-one alternative to entity-specific methods when the price for their creation and maintenance is deemed to high.

## 2 Methods

We now introduce (i) our approach to replace KB homonyms with a disambiguated version and (ii) our architectural enhancements to BioSyn, which together result in BELHD, our novel method for BEL.

### 2.1 Homonym disambiguation

Each entity *e* in a KB is represented with a unique ID associated with a set of names s∈S. For example in UMLS the entity C0030685 has the following names: “Patient Discharge,” “Discharge,” and “Release.” As shown in Fig. 1, this entails that there are two ways to represent the KB: (a) by entity or (b) by name. The latter requires defining a mapping $V_{\text{KB}}:S\to E$, which for a given *s* it returns its associated entity. The exact same name however can appear more than once in the KB, thus pointing to multiple entities. In this case we call the name a *homonym*: “Discharge” in our example. Formally, a name *s* is a homonym if $|V_{\text{KB}}(s)|>1$, where $\left|V_{\text{KB}}(s)\right|$ is the number of entities *s* maps to.

As mentioned in Section 1, homonyms severely limit the usability of name-based methods for linking. To address this issue, in BELHD, we modify how the KB is represented. For our approach we draw inspiration from Wikipedia, where article titles that would otherwise be homonymous are disambiguated via additional information in parentheses (https://en.wikipedia.org/wiki/Wikipedia:Disambiguation). Similarly, we expand every homonym into into *n* different versions, one for each entity, each augmented with a string having distinguishing information on the entity it represents. For instance we replace “Discharge” with “Discharge (Patient Discharge)” and “Discharge (Body Fluid Discharge).” The augmentation strings are other names in the KB. The procedure used to select them assumes that the KB reports for each entity which of its associated names is the *preferred* one (usually the official name).

**Figure 2.**
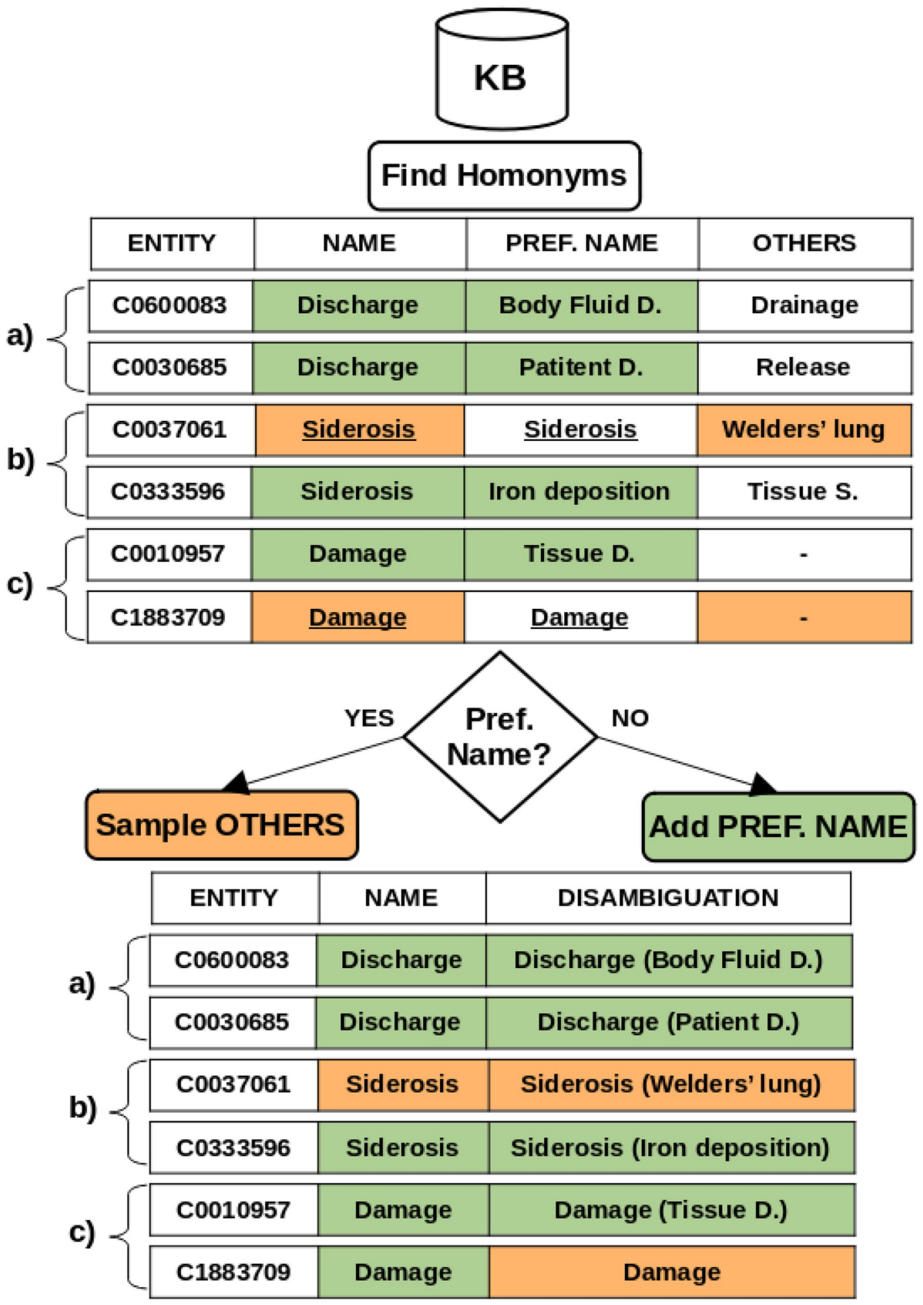
Illustration of our homonym disambiguation approach for biomedical KBs.

Our approach proceeds as follows (see Algorithm 1 in Supplementary Data C for the pseudocode). As shown in Fig. 2, we first collect all names *s* in the KB which are homonyms (see Supplementary Data B) and other names associated to the entities they refer to. (a) If the homonym is not the preferred name, we create disambiguated versions by adding the preferred name of the entities they represent. (b) If instead the homonym is itself the preferred name, we select as disambiguation string the *shortest* name (which is not the homonym) associated to the entity. This simple strategy can be replaced with a KB-specific solution if metadata is available, e.g. selecting the name’s official long form. (c) Finally, there are cases in which extending the name is not possible. This happens for homonyms which are preferred names but not do provide additional names. However, if a homonym has *n* associated entities, to ensure unique names we only need to create *n−*1 disambiguated versions, with the unmodified one acting as the default *meaning*.

#### 2.1.1 Cross-species homonyms

The approach described above is valid for any biomedical KB whose entities specify a preferred name. However, in BEL there exists a special class of homonyms which requires an additional step for complete disambiguation. Specific to Gene and Cell line, these are cross-species homonyms (For a general discussion of ambiguity in gene and cell line naming see Wei and Kao (2011) and Otto *et al.* (2017), respectively.). For instance, in NCBI Gene “*α*2microglobulin” can refer both to the human and cattle gene. As both genes have “A2M” as preferred name, our approach would generate two still identical “*α*2microglobulin (A2M).” Cross-species homonyms play such a crucial role for these entity types that the corresponding KBs (NCBI Gene and Cellosaurus) always report for each entity the species as well, in form of NCBI Taxonomy entities. For example, NCBI Gene for the human “A2M” reports 9606 (“human”) while for the cattle one 9913 (“cattle”). Therefore for these KBs, in addition to our first procedure (resolving intra-species homonyms), we identify all cross-species homonyms (see Supplementary Data B) and generate disambiguated versions with the species name from NCBI Taxonomy. If a name is both an intra- and cross-species homonym, it will have both disambiguation strings. For example, “A2M” is also a secondary name for the human gene “IGHA2,” so our approach will generate both “A2M (*α*2microglobulin, human)” and “A2M (IGHA2, human).”

### 2.2 Biomedical entity linking with homonym disambiguation

Before introducing our enhancements to BioSyn we first review its key aspect: the objective function. Sung *et al.* (2020) observe that having a name-based search space entails that there are potentially *multiple* valid candidates for a mention *m*. Therefore, they propose to train BioSyn as follows. For a given mention *m*, BioSyn uses dense retrieval to fetch a set of candidate names $C=\{c_{i},\ldots ,c_{n}\}$ from the (pre-encoded) KB (For simplicity, with a slight abuse of notation, we use *m* and *c_i_* to refer both to the strings and their embeddings.). The probability of each *c_i_* to be a correct link for *m* is defined as:

(1) $$P(c_{i}|m)=\frac{\;\text{exp}(\text{sim}(m,c_{i}))}{\sum_{j=1}^{\left|C\right|}\;\text{exp}\;(\text{sim}(m,c_{j}))},$$

where |C| is the size of *C* and sim is the inner product 〈·,·〉. The model is trained with the following per-mention *marginal maximum likelihood* (MML):

(2) $$l_{m}=-\text{log}\;\sum_{i=1}^{\left|C\right|}1_{\left[V_{C}(m)=V_{\text{KB}}(c_{i})\right]}P(c_{i}|m),$$

where $V_{C}:M\to E$ is a mapping returning the associated gold KB entity for a given mention m∈M, while $1_{\left[i=j\right]}\in \{0,1\}$ is an indicator function evaluating to 1 iff *i *=* j*. Intuitively speaking the objective function encourages the representations of *m* and all candidates names to be close in the dense space if they are associated the same KB entity. To improve BioSyn, we keep the same training objective and introduce the following changes (see Section 3.1.2 for the ablation study).

**Retrieval.** For simplicity, we described the BioSyn variant using only dense retrieval. The original model uses as well sparse retrieval (bi-gram TF-IDF) to fetch candidates from the KB. For BELHD, we rely exclusively on dense retrieval as the impact of sparse candidates in BioSyn is minimal.

**Context.** In contrast to BioSyn, BELHD leverages contextual information. For this we mark mentions boundaries with two special tokens: [S] and [E]. To obtain a single mention embedding we use mean pooling over [S] and [E].

**Projection head.** To increase search speed, we introduce a projection head parametrized by a weight matrix $W\in R^{h\times p}$ applied to *both candidates and mentions* embeddings, where *h* and *p* are the original and the projection head size, respectively. Reducing the embeddings dimension reduces as well the size of the index.

**Figure 3.**
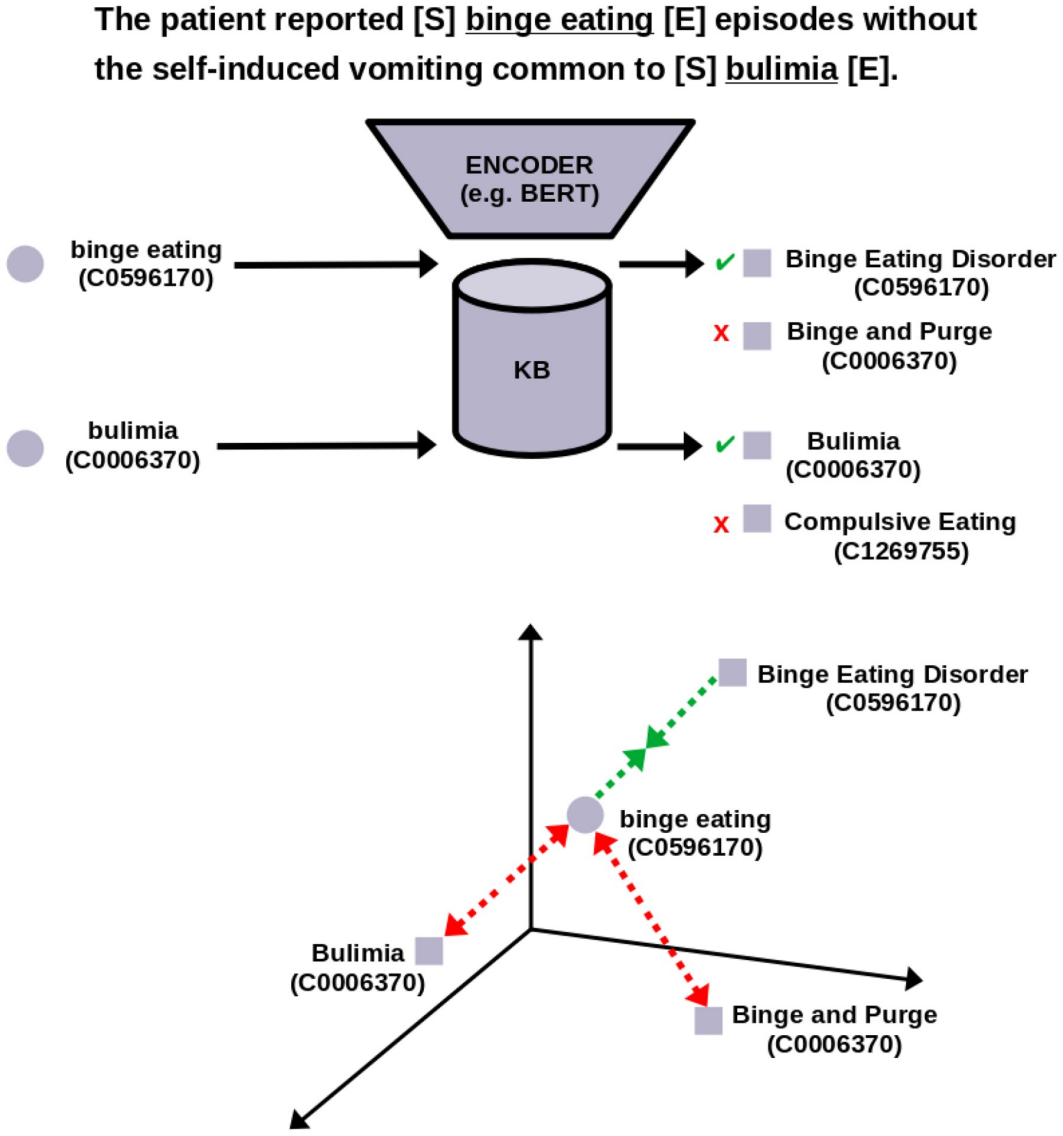
Overview of *candidate sharing*. Mentions of *binge eating* and *bulimia* co-occur presenting similar contexts, which makes them hard to distinguish. By sharing candidates we can directly force the model to consider “Bulimia” an incorrect candidate for “binge eating”.

**Candidate sharing.** During training BioSyn uses *iterative candidate retrieval*, i.e. at the end of each epoch the name embeddings in the KB are recomputed with the updated model parameters and a new set *C* for each *m* is retrieved. As a result, *C* will contain KB names that the model consider most similar to *m*. This has the benefit of increasing the chance of *C* containing *hard negatives*, i.e. KB names similar in surface form to *m*, but pointing to different entities, which push the model towards better embeddings (Gillick *et al.* 2019). Complementary to this intuition, we note that, as co-occurring mentions are often related (Chen *et al.* 2021), mentions of related entities may present similar contexts, being thus hard to differentiate. For instance, in Fig. 3 we see that “binge eating” is linked to “Binge Eating Disorder” (C0596170) and not “Bulimia” (C0006370) despite presenting words typical of mentions of the latter (“vomiting”). Importantly, “Bulimia” is also mentioned in the same document, specifically to distance “binge eating” from it. Therefore, we hypothesize that by *sharing candidates* among all mentions in a document, we can push the model to learn relevant patterns to differentiate among related entities presenting similar contexts, thus enhancing the overall training signal. In our example for instance we can directly force the model to consider “Bulimia” an incorrect candidate for “binge eating.” For this, similar to De Cao *et al.* (2021), we encode the entire input text with all of its mentions. While they use a Longformer (Beltagy *et al.* 2020) to support long sequences, we keep a BERT-based architecture (The clinical Longformer provided by Li *et al.* (2023) performed poorly on preliminary experiments.). To overcome the maximum sequence length we split each text into sentences and treat them as a single mini-batch. Then for each mention $m_{i}\in M=\{m_{i}\cdots m_{n}\}$ we create a pool of candidates *C_i_*. Half of *C_i_* is generated via iterative candidate retrieval (as in BioSyn). The other half instead is selected from $\overset{\left|M\right|}{\underset{\overset{j=1}{j\neq i}}{\cup }}C_{j}$, that is, we include candidates of other mentions *m_j_* different from those of *m_i_*. Specifically, we choose those candidates which are most similar to *m_i_*, according to their similarity (inner product). If $1_{\left[V_{C}(m_{i})=V_{\text{KB}}(c_{j})\right]}=0$, i.e. they have the same KB identifier, these are (potentially hard) negatives, positives otherwise.

### 2.3 Evaluation protocol

**Task:** We formulate BEL as predicting an entity e∈E from a KB given a document *d* and a pair of start and end positions $\langle m_{s},m_{e}\rangle$ indicating a span in *d* (the entity mention *m*). In all experiments we use in-KB (Röder *et al.* 2018) gold mentions. That is, after prediction, each *m* is associated to a KB entity and $\langle m_{s},m_{e}\rangle$ is given. **Corpora and KBs:** In all experiments we rely on BELB to access corpora and KBs. As a standardized benchmark, BELB removes confounding factors such as differences in preprocessing and KB versions. BELB consists of 10 corpora linked to 6 KBs covering 5 entity types [We exclude the Variant corpora as currently no neural method can handle its extremely large KB (1T entities).], which allows for an evaluation more comprehensive than the one reported in previous studies (see Supplementary Data B for details). Following previous work (Garda *et al.* 2023) for NCBI Gene we use the subsets determined by the species of the genes in GNormPlus and NLM-Gene (see Supplementary Data D). This reflects a common real-world use case, since often only a specific subset of species is relevant for linking (e.g. human and mouse). Finally, unlike Sung *et al.* (2020) we do not include mentions of the training corpus with their gold entity in the KB. This is because such KB expansion makes the model performance corpus-specific, while in real-world applications the model is used on arbitrary collections of documents.

## 3 Results

### 3.1 Homonym disambiguation

The effectiveness of our approach in disambiguating homonyms is measured by its success rate, i.e. the ratio between homonyms which after expansion are no longer homonyms and the original ones in the KB. From Table 1 we see that our approach can disambiguate virtually all homonyms in all six KBs (see Supplementary Data D for NCBI Gene subsets).

**Table 1.** Number of names and homonyms (% relative to names) categorized by cases (see Section 2.1) in BELB KBs.

	CTD diseases (Disease)	CTD chemicals (Chemical)	Cellosaurus (Cell line)	NCBI gene (Gene)	NCBI taxonomy (Species)	UMLS
Entities	13 188	175 663	144 568	42 252 923	2 491 364	3 464 809
Names	88 548	451 410	251 747	105 570 090	3 783 882	7 938 833
Homonyms	349 (0.39%)	2 (<0.1%)	8070 (3.21%)	56 597 279 (53.61%)	1422 (0.04%)	164 154 (2.07%)
pref. name	1		502	822 103	27	12 530
other	348	2	2582	20 965 362	1395	151 624
cross-species			5416	56 265 683		
Success rate	100%	100%	100%	>99.99% (1054)	100%	>99.99% (39)
Avg. name length	26.68 (+0.16)	28.99 (-)	12.29 (+0.44)	36.07 (+14.66)	28.06 (+0.01)	40.18 (+0.66)

The success rate is the ratio between homonyms which after augmentation are no longer homonyms and the original amount. Number of failures, i.e. names that are still homonyms (duplicates) after HD is reported in parenthesis. We report as well the average name length before and after (in brackets) disambiguation.

The failure cases are KB names having the same surface form *after* our approach extends them with distinguishing information. These are not to be confused with instances of case (c) in Section 2.1, i.e. where a preferred name is a homonym but has no alternative names. We find that these are caused by a combination of two factors. The first is related to the quality of the KBs. UMLS and NCBI Gene are meta-KBs, i.e. they integrate entities from multiple KBs. In few cases, this causes them to have two distinct entities having however little to no difference in terms of names. The second is using the shortest alternative name as disambiguation string when a homonym is also the entity’s preferred name. For example, in UMLS C0003663 and C0020316 have almost identical list of associated names. C0003663’s preferred name is “Aquacobalamin,” having as secondary name “Hydroxocobalamin.” However, “Hydroxocobalamin” is also C0020316’s preferred name, whose shortest alternative name is “Aquacobalamin,” thus our disambiguation generates two “Hydroxocobalamin (Aquacobalamin).” Finally, we note that after HD two important KB characteristics change: size, as homonyms are not considered as duplicate anymore and (b) average name length, as we add a disambiguation string to each homonym (see Supplementary Data I for impact on inference speed).

### 3.2 Biomedical entity linking with homonym disambiguation

From Table 2 we see that BELHD is the overall best approach, outperforming all methods considered in 6 out of 10 corpora, with GenBioEL as second. Our results show that name-based approaches generally outperform those relying on entity representations, which is in line with findings in the general domain (Milich and Akbik 2023). We hypothesize that this is due to the fact that name-based dense methods are more sample-efficient, as they can directly leverage surface similarities between mentions and KB names, while the entity-based one requires more training data to optimize entity representations.

**Table 2.** Performance of all models on BELB corpora (test set).

	**CTD diseases** (Disease)		**CTD chemicals** (Chemical)		**Cellosaurus** (Cell line)	**NCBI gene** (Gene)		**NCBI taxonomy** (Species)		UMLS
	NCBI disease	BC5CDR (D)	BC5CDR (C)	NLM-Chem	BioID	GNormPlus	NLM-Gene	S800	Linnaeus	MedMentions
*Entity-based*										
**arboEL**^a^	80.00	84.87	87.40	71.76	95.02	34.64	29.96	78.62	74.97	68.67
**KRISSBERT**^a^^,^^b^	82.80	85.0	**95.10**	-	-	-	-	-	-	61.30
*Name-based*										
**BioSyn**	79.90	84.83	84.57	70.35	80.79	OOM	OOM	82.79	88.60	OOM
+ HD	79.90	$84.23_{-0.60}$	$85.00_{+0.43}$	$71.31_{+0.96}$	$81.60_{+0.9}$	OOM	OOM	$81.23_{-1.56}$	**88.81**_+0.21_	OOM
**GenBioEL**	82.71	88.29	94.60	75.00	94.79	6.80	2.89	88.27	76.92	41.16
+ HD	83.02_+0.31_	$88.20_{-0.09}$	$94.15_{-0.45}$	$74.10_{-0.90}$	${\underline{96.30}}_{+1.51}$	${\underline{66.08}}_{+59.28}$	**66.43**_+63.54_	**89.96**_+1.69_	$77.62_{-0.30}$	$64.59_{+23.43}$
**BELHD (ours)**	**87.60**	**89.23**	92.93	**82.39**	**96.99**	**77.84**	59.03	84.35	81.89	**70.58**

a Without cross-encoder reranking.

b Authors provide code only for the “supervised” variant (https://huggingface.co/microsoft/BiomedNLP-KRISSBERT-PubMed-UMLS-EL), i.e. entity prototypes are based on train/development mentions. As this variant cannot link zero-shot entities we report results from Table 7 in Zhang *et al.* (2022) for a fair comparison.

Bold and underlined indicate best and second best score, respectively. HD, homonym Disambiguation (Section 2.1); OOM, out-of-memory (>200GB).

Importantly, we note that our HD solution can be used with any name-based model. Notably, when equipped with HD, GenBioEL’s performance increases by 63.54pp on the homonym-rich NLM-Gene, outperforming all other methods, including BELHD. This can be attributed to the fact that the corpus was specifically created to test models on cross-species homonyms (Islamaj *et al.* 2021). Key to success on the task is contextual information, primarily in form of species mentions. While GenBioEL processes the entire text, BELHD uses sentences (see Section 2.2), thus being unable to access species information if it does not occur in the same sentence as the gene mention. Finally, we see that HD as minimal to no effect in BioSyn, since it does not use context. BioSyn is however the best model for the Linnaues corpus. This can be explained by the fact that Linnaeus was created specifically for the development of dictionary-based approaches (Luoma *et al.* 2023), giving a strong advantage to methods using string-matching like BioSyn.

#### 3.2.1 Ablation study

Table 3 reports our ablation study of BELHD improvements over BioSyn (see Section 2.2). We see that the most important component is HD, which is critical for a homonym-rich entity type like Gene, while the use of contextual information is second. The third strongest improvement is brought by *candidate sharing*. This confirms that leveraging the relatedness of co-occurring mentions enhances training signal improving overall results. Finally, the projection head, despite reducing the embedding dimensionality, does not hurt performance.

**Table 3.** Ablation study of improvements over BioSyn introduced in BELHD (see Section 2.2).

	NLM-Gene
**BELHD (ours)**	59.03
- HD	$6.67_{-52.8}$
- context	$32.72_{-26.31}$
- candidate sharing	$56.83_{-2.20}$
- projection head	$58.74_{-0.29}$

#### 3.2.2 *Ad-hoc* solutions for HD

Here, we compare *ad-hoc* methods to handle homonyms with our general HD approach on the corpora most affected by homonyms. We perform abbreviation resolution (AR) with Ab3P (replacing abbreviations with their long form) and retrain all models. Second, we identify and assign species to gene mentions with SpeciesAssignment (SA) and filter predictions accordingly. As AR resolves difficult mentions which are not necessarily affected by homonyms (see Supplementary Section A) we include the entity-based arboEL in the comparison. As SA is specific to name-based methods, for arboEL we include species name in entity representations as proposed by Kartchner *et al.* (2023). From Table 4 we see that HD delivers the best results across corpora. It significantly outperforms AR, confirming that addressing a wider range of homonyms is critical. Interestingly, HD outperforms the highly specialized SA approach as well. We argue that this is due to species information not being always explicitly expressed (Wei *et al.* 2012), upon which SA relies. HD instead is more versatile, allowing the model to *learn* useful contextual patterns beyond explicit species mentions.

**Table 4.** Effect of different strategies to handle homonyms.

	UMLS	**NCBI Gene** (Gene)	
	MedMentions	GNormPlus	NLM-Gene
**arboEL**	68.67	34.64	29.96
+ AR	$68.85_{+0.18}$	$35.66_{+1.02}$	$30.30_{+0.34}$
+ Species^a^		**47.83_+13.19_**	**40.82_+10.86_**
**GenBioEL**	41.16	6.80	2.89
+ AR	$42.01_{+0.85}$	$7.67_{+0.87}$	$3.48_{+0.59}$
+ SA		$65.70_{+58.90}$	$61.78_{+58.89}$
+ HD	**64.59_+23.43_**	**66.08_+59.28_**	**66.43_+63.54_**
**BELHD w/o HD**	57.23	13.90	6.67
+ AR	$59.50_{+2.27}$	$15.98_{+2.08}$	$6.85_{+0.18}$
+ SA		$43.67_{+29.77}$	$42.58_{+35.91}$
+ HD	**70.58_+13.35_**	**77.84_+63.94_**	**59.03_+52.35_**

a Include species name into entity representation (Kartchner *et al.* 2023).

HD, homonym disambiguation (ours); AR, abbreviation resolution (Sohn *et al.* 2008); SA, species assignment (Luo *et al.* 2022b). **Bold **values indicate best score.

## 4 Discussion

Our experiments show that BELHD outperforms all methods in 6 out of 10 BEL corpora. We stress that, due to the high computational resources necessary to train BEL models, we retrain all models on BELB with code and hyperparameters provided by the authors. It is therefore possible that optimizing them may result in better numbers. However, we believe that this setting is the best approximation to fairly compare across methods since BEL studies present stark differences in preprocessing, corpora, and experimental setups making comparison based on published numbers problematic (Garda *et al.* 2023). We note as well that we avoid corpus- or KB-specific pre-trained weights. This is because, as noted by Milich and Akbik (2023), different methods use different different data, ultimately impairing direct comparison. It is therefore possible that GenBioEL, with its *KB-Guided Pre-training* may achieve higher results. Additionally, we note that for neural approaches it is a common practice to train a second model to rerank the subset of entities selected by a candidate generator (French and McInnes 2022). In our study however we intentionally skip this refinement as it is a further enhancement entirely *dependent* on the results of the generator: its performance is by design bound by the recall of the generator.

Secondly, we show that HD is a general solution allowing name-based methods to return unique linking decision, which outperforms existing *ad-hoc* approaches like AR and species assignment. HD is based on the assumption that a KB must specify a preferred name for every entity. Though, to the best of our knowledge, virtually all biomedical KBs meet this assumption, this entails that HD is not strictly KB-agnostic. By being fully-automatic and having a single minimal assumption, HD sets itself apart from existing strategies which modify the KB to reduce its ambiguity. This is because these require either (i) human intervention, e.g. the assignment of semantic types (https://lhncbc.nlm.nih.gov/ii/tools/MetaMap/documentation/SemanticTypesAndGroups.html) in UMLS, or (ii) concept descriptions/summaries (Schijvenaars *et al.* 2005, Procopio *et al.* 2023), which in most KBs are not available or present only for a limited subset of concepts (Zhang *et al.* 2022). Furthermore, as some biomedical KBs have entities with almost identical list of names, our approach, though greatly reducing their number, does not completely resolve all homonyms (see Section 2.1). This could be mitigated by using more sophisticated strategies to select the disambiguation string, e.g. using the least similar name determined by Levenshtein distance, which we leave as future work.

Finally, we compare BELHD to a set of state-of-the-art non-neural entity-specific BEL models aggregated in PubTator3 (Wei *et al.* 2024). To this end, we replicate PubTator3 experimental setting and measure BELHD performance when applied in end-to-end entity linking, i.e. operating on mentions extracted by an entity recognition model. Results in Table 5 show that BELHD gives better performance on all entity types, partly with more than 5pp difference, except for genes. PubTator’s superior performance on this entity is probably due its usage of GNorm2 (Wei *et al.* 2023), a manually tailored method using curated dictionaries specifically crafted for the task (see Supplementary Data F). We note that differences in the KBs and their applied versions used by the evaluations may affect these results (Garda *et al.* 2023). In this particular comparison, however, we expect them to be negligible, as both methods use the same KBs in fairly recent versions. As our contributions focus on neural approaches, we leave as future work the investigation of (i) other non-neural methods like Personalized Page Rank (Lamurias *et al.* 2019) and (ii) hybrid systems (Sung *et al.* 2022).

**Table 5.** Comparison with PubTator3 on end-to-end entity linking (entity recognition + linking).

	Disease	Chemical	Cell line	Gene	Species
**PubTator3**	79.17	81.92	80.85	84.63	95.20
**BELHD (ours)**	84.32	83.93	87.06	80.50	96.55

We report document-level F1 score on the test set of BioRED (Luo *et al.* 2022a). Results of PubTator3 are those reported in Wei *et al.* (2024). Linking is performed on mentions identified by AIONER (Luo *et al.* 2023).

## 5 Conclusion

We highlight how homonyms in biomedical KBs significantly impact performance of BEL methods returning KB names as predictions. We introduce BELHD, a novel BEL approach based on BioSyn (Sung *et al.* 2020) outperforming all the comparable methods considered in 6 out of 10 corpora. We show that its primary feature HD is a general solution, which can be used to improve results in other name-based methods as well.

## Supplementary Material

btae474_Supplementary_Data

## Data Availability

Our code and data are available at: https://github.com/sg-wbi/belhd.
