# Supplementary material for: BELHD: improving biomedical entity linking with homonym disambiguation
Source: Bioinformatics. 2024 Jul 27;40(8):btae474. doi: 10.1093/bioinformatics/btae474 (PMC11310454; doi:10.1093/bioinformatics/btae474)
Supplement: btae474_Supplementary_Data [file btae474_supplementary_data.pdf]

Journal Title Here, 2022, 1–8
doi: DOI HERE
Advance Access Publication Date: Day Month Year
Paper

Supplementary data for

BELHD: Improving Biomedical Entity Linking with Homonym Disambiguation

Samuele Garda[1,*] and Ulf Leser[1,*]

[1]Computer Science, Humboldt-Universität zu Berlin, Rudower Chaussee 25, 12489, Berlin, Germany

*To whom correspondence should be addressed.

FOR PUBLISHER ONLY Received on Date Month Year; revised on Date Month Year; accepted on Date Month Year

Abstract

Motivation Biomedical entity linking (BEL) is the task of grounding entity mentions to a given knowledge base (KB). Recently, neural name-based methods, system identifying the most appropriate name in the KB for a given mention using neural network (either via dense retrieval or autoregressive modeling), achieved remarkable results for the task, without requiring manual tuning or definition of domain/entity-specific rules. However, as name-based methods directly return KB names, they cannot cope with homonyms, i.e. different KB entities sharing the exact same name. This significantly affects their performance for KBs where homonyms account for a large amount of entity mentions (e.g. UMLS and NCBI Gene).
Result We present **BELHD** (**B**iomedical **E**ntity **L**inking with **H**omonym **D**isambiguation), a new name-based method that copes with this challenge. BELHD builds upon the BioSyn (Sung et al., 2020) model with two crucial extensions. First, it performs a pre-processing of the KB during which it expands homonyms with an specifically constructed disambiguating string, thus enforcing unique linking decisions. Second, it introduces *candidate sharing*, a novel strategy that strengthens the overall training signal by including similar mentions from the same document as positive or negative examples, according to their corresponding KB identifier. Experiments with ten corpora and five entity types show that BELHD improves upon current neural state-of-the-art approaches, achieving the best results in six out of ten corpora with an average improvement of 4.55pp recall@1. Furthermore, the KB preprocessing is orthogonal to the prediction model and thus can also improve other neural methods, which we exemplify for GenBioEL (Yuan et al., 2022), a generative name-based BEL approach.
Availability and Implementation: The code to reproduce our experiments can be found at: `https://github.com/sg-wbi/belhd`.
Contact: leser@informatik.hu-berlin.de
Supplementary Information: Supplementary data are available at Bioinformatics online.

A. Homonyms

In case of name-based systems, homonyms fundamentally disrupt linking: mentions linked to a homonym s cannot be mapped to a unique KB entity. Determining the extent of the issue, i.e. how many mentions homonyms account for, is however non-trivial. This is because the number of mentions linked to homonyms depends on the specific model. For instance, for the mention "discharge" in Fig. 1, it is possible for a name-based system to return "Patient Discharge". However, due to the high similarity between surface forms, we expect most current models to rank "Discharge" higher. We obtain an *approximate* estimate by considering mentions to be affected by homonyms if their gold KB entity has an associated name (a) which is a homonym and (b) whose surface form is highly similar to the

© The Author 2022. Published by Oxford University Press. All rights reserved. For permissions, please e-mail: journals.permissions@oup.com

**BELB: Biomedical Entity Linking Benchmark** (Garda et al., 2023)		
KB (entity type)		Homonyms
Corpus	Affected mentions	
CTD Diseases (Disease)		0.39%
NCBI Disease	1.25% (12 / 960)	
BC5CDR (D)	0.18% (8 / 4,363)	
CTD Chemicals (Chemical)		>0.1%
BC5CDR (C)	0% (0 / 5,334)	
NLM-Chem	0% (0 / 11,514)	
Cellosaurus (Cell line)		3.21%
BioID	3.47% (30 / 864)	
NCBI Gene (Gene)		53.61%
GNormPlus	68.84% (2,218 / 3,222)	
NLM-Gene	66.1% (1,804 / 2,729)	
NCBI Taxonomy (Species)		0.04%
Linnaeus	0% (0 / 1,430)	
S800	0% (0 /767)	
UMLS		2.07%
MedMentions (st21pv)	26.41% (10,602 / 40,143)	

Table 1. Relative number of homonyms and approximate estimate of the links they account for in BELB (test set).

mention's one. Concretely, we consider two strings[1] to be highly similar if their normalized Levenshtein distance[2] (Marzal and Vidal, 1993) is one.

In Table 1 we report this estimate for corpora in BELB Garda et al. (2023), a BEL benchmark comprising ten commonly used corpora linked to six KBs. Though some corpora have no such cases, we see that despite being only ∼2% of UMLS names (see Table 1 for exact counts), homonyms may account for up to 26% of the links in the widely used `MedMentions`, imposing an upper bound to the performance of name-based methods. The amount rises to >60% for genes, for which homonyms are a well known aspect (Wei and Kao, 2011; Kartchner et al., 2023) as the same gene can be found in multiple species (see Section 2.1.1).

B. Identify homonyms

Listing 1 Schema used in BELB to store biomedical KBs.

```
CREATE TABLE kb(
    uid INTEGER PRIMARY KEY,
    -- entity label
    identifier INTEGER NOT NULL,
    -- pref. name (0), abbr. (1), ...
    description INTEGER NOT NULL,
    name TEXT NOT NULL,
    -- NCBI Taxonomy entity
    species INTEGER DEFAULT NULL
)
```

In Listing 1 we present the unified schema provided in BELB used to store all biomedical KB. Each name is associated to an identifier (entity label) and a description, i.e. whether it is e.g. the preferred name or the abbreviated form. In case of KBs having cross-species homonyms (see Section 2.1.1) BELB stores the name of the associated species entity coming from NCBI Taxonomy.

Listing 2 shows how using BELB we generate the set of homonyms and cross-species homonyms. In case of KBs having cross-species homonyms (see Section 2.1.1) the group by of the first query includes "species" to ensure that the homonyms belong to the same species (intra-species), e.g. "BRI3" can be either NCBI Gene 81618 or 25798, both however are *human* genes. The second query instead specifically identifies only cross-species homonyms, i.e. it constraints names to have different associated species.

[1] We preprocess mentions and KB names by lowercasing and removing all non alphanumeric characters.

[2] We use the implementation provided by `https://github.com/maxbachmann/RapidFuzz`.

Listing 2 Example SQL queries to compute set of homonyms with BELB KBs.

```
-- Homonyms
SELECT name FROM kb GROUP BY name,species HAVING count(*)>1

-- Cross-species homonyms
SELECT name FROM kb GROUP BY name HAVING count(*)>1 AND COUNT(DISTINCT(species))>1;
```

C. Pseudocode for Homonym Disambiguation procedure

Algorithm 1 Pseudocode our disambiguation approach.

```
Require: H                                   ▷ Pre-computed set of homonyms
Require: E                                   ▷ Entities
for each e ∈ E do
    S ← get_names(e)
    for each s ∈ S do
        if s ∈ H then
            p ← get_preferred_name(S)
            if s = p then
                ▷ s.t. s != p
                d ← get_longest(s, S)
            else
                d ← p
            end if
            s ← concatenate(s, d)
        end if
    end for
end for
```

In Algorithm 1 we present the pseudocode of our approach to resolve homonyms in biomedical KBs present in Section 2.1. The pseudocode for the cross-species procedure described in Section 2.1.1 can be easily derived from it.

D. Corpus-specific NCBI Gene subsets

	NCBI Gene (Gene)	
	`GNormPlus`	`NLM-Gene`
Names	2,455,772	2,913,456
Homonyms	1,163,255 (47.37%)	1,479,719 (50.79%)
- pref. name	33,919	35,032
- other	323,824	39,1290
- cross-species	1,094,531	1,410,006
Success rate	>99% (520)	>99% (523)
Avg. name length	28.74 (+8.34)	29.94 (+9.06)

Table 2. Equivalent to Table 1 for NCBI Gene corpus-specific subsets.

For the NCBI Gene subsets determined by the species of the genes in `GNormPlus` and `NLM-Gene` (see Section 2.3) we report in Table 2 the number of homonyms and the success rate of our disambiguation approach. In Table 3 we report the NCBI Gene subsets determined by the species (NCBI Taxonomy entities) of the gene mentions in `GNormPlus` and `NLM-Gene`.

E. Models and training details

Here we report training details for all models considered in our study. We stress that we retrain all models on BELB with the code provided by the original authors (see Table 4 for links to implementations). All experiments were performed on two NVIDIA A100 GPUs.

NCBI Taxonomy		
Entity	Name	Corpora
3055	Chlamydomonas reinhardtii	NLM-Gene
3702	thale cress	GNormPlus,NLM-Gene
3847	soybean	GNormPlus
4896	fission yeast	GNormPlus,NLM-Gene
6239	Caenorhabditis elegans	GNormPlus,NLM-Gene
6956	European house dust mite	NLM-Gene
7227	fruit fly <Drosophila melanogaster>	GNormPlus,NLM-Gene
7955	zebrafish	GNormPlus,NLM-Gene
8355	African clawed frog	GNormPlus,NLM-Gene
8364	tropical clawed frog	GNormPlus,NLM-Gene
9031	chicken	GNormPlus,NLM-Gene
9606	human	GNormPlus,NLM-Gene
9615	dog	NLM-Gene
9823	pig	GNormPlus,NLM-Gene
9913	cattle	GNormPlus,NLM-Gene
9940	sheep	NLM-Gene
9986	rabbit	GNormPlus,NLM-Gene
10029	Chinese hamster	NLM-Gene
10089	Ryukyu mouse	NLM-Gene
10090	house mouse	GNormPlus,NLM-Gene
10116	Norway rat	GNormPlus,NLM-Gene
10298	Herpes simplex virus type 1	GNormPlus
11676	Human immunodeficiency virus 1	GNormPlus,NLM-Gene
11709	Human immunodeficiency virus 2	NLM-Gene
11908	Human T-cell leukemia virus type I	GNormPlus
41856	Hepatitis C virus genotype 1	GNormPlus
51031	New World hookworm	NLM-Gene
81972	Arabidopsis lyrata subsp. lyrata	NLM-Gene
333760	Human papillomavirus type 16	GNormPlus
511145	Escherichia coli str. K-12 substr. MG1655	GNormPlus
559292	Saccharomyces cerevisiae S288C	GNormPlus,NLM-Gene
2886926	Escherichia phage P1	NLM-Gene

Table 3. NCBI Gene subsets determined by the species (NCBI Taxonomy entities) of the gene mentions in GNormPlus and NLM-Gene

	Implementation (link)
arboEL	https://github.com/dhdhagar/arboEL
GenBioEl	https://github.com/Yuanhy1997/GenBioEL
BioSyn	https://github.com/dmis-lab/BioSyn

Table 4. Implementation links of the biomedical entity linking models used in our experiments.

BioSyn uses the default hyper-parameters provided by the authors. Unlike in the original study, we exclusively train on the train split of corpora (no development). Total amount of parameters: 110M.
GenBioEl uses different values for learning rate and warmup steps for `NCBI Disease` and `BC5CDR`. We cannot perform a full hyper-parameter search for each corpus, and therefore select the values that work best for both corpora, i.e. a learning rate of $1e-5$ and 500 warmup steps. Total amount of parameters: 400M.
arboEL's inference procedure is parametrized by the number of k nearest neighbor used to construct the graph (determining which pairs of nodes are connected). The implementation provided by the authors runs the inference trying different $k \in \{0, 1, 2, 4, 8\}$. For fair comparison with other models, we do not perform any hyperparameter optimization and hence report the score for $k = 0$. Total amount of parameters: 110M.
BELHD keeps all BioSyn hyper-parameter besides (i) the number of training epochs which we increase from 10 to 20 and the number of candidates k for each mention m_i, which we set to 32: 16 mention-specific and 16 from candidate sharing (see Section 2.2) Like in Biosyn, the KB is re-encoded at the and of each training epoch to keep the name embeddings consistent with the updated model parameters Guu et al. (2020). The only exception is `MedMentions`, for which, due to its large size, we use 10 epochs and re-encode the KB every 1000 steps. The dimensionality of the projection head is set to 128. In order to fit the entire text unit at once, we split it into sentences (with

segtok[3]) and treat it as a single mini-batch, using on gradient accumulation to achieve a larger batch size, which we set to 8. We rely on FAISS Johnson et al. (2019) for efficient exact maximum inner product search. Total amount of parameters: 110M (projection head has 1e4 parameters).

F. PubTator3

	NER	Linking	KB
Disease		TaggerOne † (Leaman and Lu, 2016)	CTD Diseases (Davis et al., 2023)
Chemical		NLM-Chem (Islamaj et al., 2021a)	MeSH (Lipscomb, 2000)
Cell Line	AIONER	TaggerOne † (Leaman and Lu, 2016)	Cellosaurus (Bairoch, 2018)
Gene		GNorm2 (Wei et al., 2023)	NCBI Gene (Brown et al., 2015)
Species		GNorm2 (Wei et al., 2023)	NCBI Taxonomy (Scott, 2012)

Table 5. Overview of the tools used by PubTator3. † Improved version introduced in (Wei et al., 2024)

PubTator3 (Wei et al., 2024) is a web-interface which aggregates state-of-the-art tools for biomedical text mining. In Table 5 we provide an overview of all tools deployed in PubTator3. We note that the entity linking (normalization) tools are non-neural entity-specific systems. The KBs used for normalization are gathered from inspecting the original publication of each tool.

	Disease			Chemical			Cell Line			Gene			Species		
	P	R	F1	P	R	F1	P	R	F1	P	R	F1	P	R	F1
**PubTator3**	75.33	83.43	79.17	83.26	80.63	81.92	76.00	86.36	80.85	90.60	79.41	84.63	93.97	96.46	95.20
**BELHD**	80.34	88.72	84.32	79.50	88.89	83.93	83.97	90.38	87.06	76.20	85.32	80.50	95.47	97.64	96.55

Table 6. Comparison with PubTator3. We report document-level precision (P), recall (R) and F1 score on the test set of the BioRED corpus (Luo et al., 2022). PubTator3's results are those reported in (Wei et al., 2024). Linking is performed on mentions identified by the AIONER (Luo et al., 2023) model.

In Table 6 we report the document-level results on the BioRED corpus (Luo et al., 2022) of PubTator3 compared with our BELHD model. Mentions are extracted by the AIONER (Luo et al., 2023) model (the version using PubmedBERT-CRF (Luo et al., 2022) as backbone pre-trained language model). We highlight that, as noted by Garda et al. (2023), differences in the KBs (and their versions) used by the two methods may affect their results. In this case however we expect them to be only minor, as PubTator3 and BELHD use almost the same KBs and their fairly recent versions.

G. BELHD: disambiguation example

H. Biomedical Entity Linking Benchmark

BELB is a biomedical entity linking benchmark introduced by Garda et al. (2023). It provides access to 10 corpora linked to six knowledge bases and spanning five entity types: Gene, Disease, Chemical, Species and Cell lines: see Table 7 and Table 8 for an overview of the KBs and corpora, respectively. The key feature of BELB is its standardized preprocessing of corpora and KBs, offering tight integration between the two. This makes it a standardized testbed which removes confounding factors such as differences in preprocessing and KB versions. All corpora consists of biomedical publications in English. For a detailed description and license information of each corpus and KB we refer the reader to the original publication. We note as well that the S800 corpus (Pafilis et al., 2013) has recently been superseded by the S1000 (Luoma et al., 2023) corpus.

I. Inference speed on KB with and without disambiguation

A disambiguated KB does differ from the original one in two important aspects: (a) size, because homonyms are not considered as duplicate anymore and (b) average name length, as we add a disambiguation string to each homonym. Therefore we report the average inference speed of BELHD using the two different versions. For the comparison we use the test set of `NLM-Gene` which is linked to `NCBI Gene`, the KB most affected by homonym disambiguation. Results in Table 9 show that with the disambiguated KB inference speed is,as expected, slower. However, the combined effect of increased size and average name length is minimal (∼0.7 second more per abstract).

[3] https://github.com/fnl/segtok

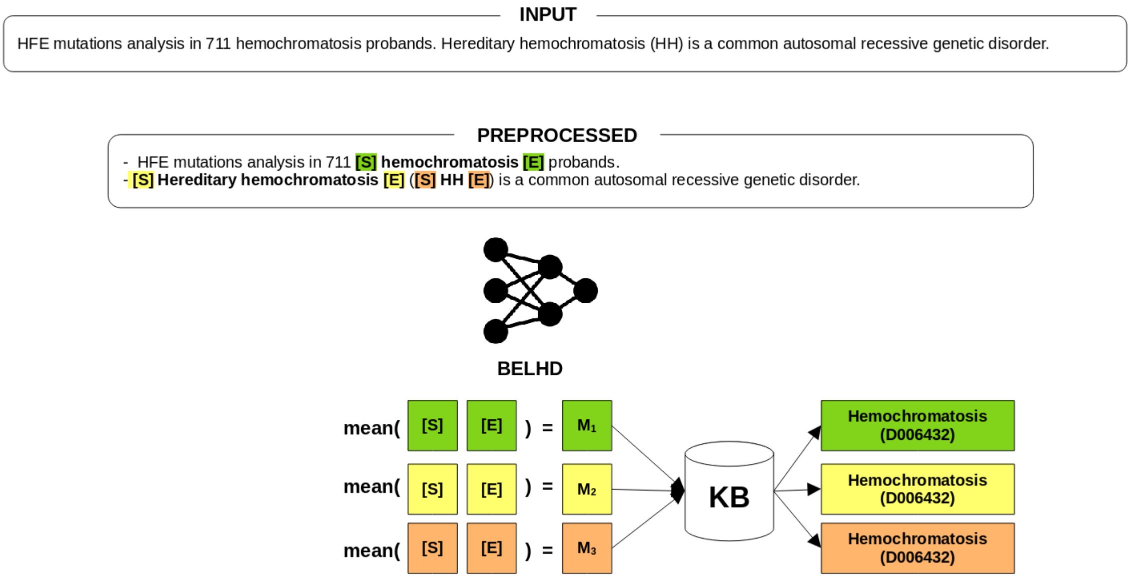

Fig. 1: Example of obtaining linking predictions with BELHD. Given an input text BELHD (a) splits it into sentences and (b) adds special tokens (**[S]**, **[E]**) to mark the boundaries of all mentions (mention boundaries must be provided as input). It then encodes the preprocessed sentences in one step (mini-batch) and extracts the embeddings corresponding to the special tokens. These are averaged and used as final embedding to search the encoded KB to obtain predictions (KB names).

Entity type<br>KB	Entities	Names	Avg. names per entity
Disease<br>CTD Diseases Davis et al. (2023)	13,188	88,548	6.71
Chemical<br>CTD Chemicals Davis et al. (2023)	175,663	451,410	2.56
Cell line<br>Cellosaurus Bairoch (2018)	144,568	251,747	1.74
Species<br>NCBI Taxonomy Scott (2012)	2,491,364	3,783,882	1.51
Gene<br>NCBI Gene Brown et al. (2015)	42,252,923	105,570,090	2.49
`GNormPlus` subset	703,858	2,455,772	3.48
`NLM-Gene` subset	873,015	2,913,456	3.33
UMLS<br>UMLS Bodenreider (2004)	3,464,809	7,938,833	2.29

Table 7. Overview of the KBs available in BELB according to their entity type. We report the number of entities, names and average name per entities

References

C. Arighi, L. Hirschman, T. Lemberger, S. Bayer, R. Liechti, D. Comeau, and C. Wu. Bio-ID track overview. In *BioCreative VI Challenge Evaluation Workshop*, volume 482, page 376, 2017. URL https://biocreative.bioinformatics.udel.edu/media/store/files/2018/BC6_track1_1.pdf.

A. Bairoch. The cellosaurus, a cell-line knowledge resource. *Journal of biomolecular techniques: JBT*, 29(2):25, 2018.

O. Bodenreider. The Unified Medical Language System (UMLS): integrating biomedical terminology. *Nucleic Acids Research*, 32: 267D–270, 1 2004. doi: 10.1093/nar/gkh061.

G. R. Brown, V. Hem, K. S. Katz, M. Ovetsky, C. Wallin, O. Ermolaeva, I. Tolstoy, T. Tatusova, K. D. Pruitt, D. R. Maglott, and T. D. Murphy. Gene: a gene-centered information resource at ncbi. 43(D1):D36–D42, 2015. ISSN 0305-1048. doi: 10.1093/nar/gku1055.

A. P. Davis, T. C. Wiegers, R. J. Johnson, D. Sciaky, J. Wiegers, and C. J. Mattingly. Comparative Toxicogenomics Database (CTD): update 2023. *Nucleic Acids Research*, 51:D1257–D1262, 1 2023. doi: 10.1093/nar/gkac833.

R. I. Doğan, R. Leaman, and Z. Lu. NCBI disease corpus: A resource for disease name recognition and concept normalization. *Journal of Biomedical Informatics*, 47:1–10, 2014. ISSN 1532-0464. doi: 10.1016/j.jbi.2013.12.006.

S. Garda, L. Weber-Genzel, R. Martin, and U. Leser. BELB: a biomedical entity linking benchmark. *Bioinformatics*, 11 2023. doi: 10.1093/bioinformatics/btad698.

Entity type / Corpus	Documents (train / dev / test)	Mentions (train / dev / test)	0-shot mentions
Disease			
NCBI Disease Doğan et al. (2014)	592 / 100 / 100	5,133 / 787 / 960	150 (15.62%)
BC5CDR (D) Li et al. (2016)	500 / 500 / 500	4,149 / 4,228 / 4,363	388 (8.89%)
Chemical			
BC5CDR (C)Li et al. (2016)	500 / 500 / 500	5,148 / 5,298 / 5,334	1,038 (19.46%)
NLM-Chem † Islamaj et al. (2022)	80 / 20 / 50	20,796 / 5,234 / 11,514	3,908 (33.94%)
Cell line			
BioID ‡ Arighi et al. (2017)	231 / 59 / 60	3,815 / 1,096 / 864	158 (18.29%)
Species			
Linnaeus † Gerner et al. (2010)	47 / 17 / 31	2,115 / 705 / 1,430	385 (26.92%)
S800 (Pafilis et al., 2013)	437 / 63 / 125	2,557 / 384 / 767	363 (47.33%)
Gene			
GNormPlus Wei et al. (2015)	279 / 137 / 254	3,015 / 1,203 / 3,222	2,822 (87.59%)
NLM-Gene Islamaj et al. (2021b)	400 / 50 / 100	11,263 / 1,371 / 2,729	1,215 (44.52%)
UMLS			
MedMentions (st21pv) (Mohan and Li, 2019)	2,635 / 878 / 879	122,178 / 40,864 / 40,143	8,167 (20.34%)

Table 8. Overview of the corpora available in BELB with number of documents, mentions and 0-shot mentions (mentions linked to an entity not in the train/development set). Pairing of corpora and KB is determined by the entity type. ‡ Full text ‡ Fig. captions

	NCBI Gene	
	Original	with HD
Names (size)	1,775,214	2,913,456 (+64.03%)
Avg. name length	20.88	29.94 (+9.06)
Sec. per Abstract	1.27 (±0.04)	2.02 (±0.04)

Table 9. Inference speed of BELHD using NCBI Gene with and without disambiguation. We run 3 independent runs on the NLM-Gene test set and report the average amount of seconds required to process an abstract on GPU. We report as well the relative (in brackets) increase in size (number of names) and average name length.

M. Gerner, G. Nenadic, and C. M. Bergman. Linnaeus: a species name identification system for biomedical literature. *BMC bioinformatics*, 11(1):1–17, 2010. doi: 10.1186/1471-2105-11-85.

K. Guu, K. Lee, Z. Tung, P. Pasupat, and M. Chang. Retrieval augmented language model pre-training. In H. D. III and A. Singh, editors, *Proceedings of the 37th International Conference on Machine Learning*, volume 119 of *Proceedings of Machine Learning Research*, pages 3929–3938. PMLR, 13–18 Jul 2020. URL https://proceedings.mlr.press/v119/guu20a.html.

R. Islamaj, R. Leaman, S. Kim, D. Kwon, C.-H. Wei, D. C. Comeau, Y. Peng, D. Cissel, C. Coss, C. Fisher, R. Guzman, P. G. Kochar, S. Koppel, D. Trinh, K. Sekiya, J. Ward, D. Whitman, S. Schmidt, and Z. Lu. Nlm-chem, a new resource for chemical entity recognition in pubmed full text literature. *Scientific Data*, 8, 3 2021a. doi: 10.1038/s41597-021-00875-1.

R. Islamaj, C.-H. Wei, D. Cissel, N. Miliaras, O. Printseva, O. Rodionov, K. Sekiya, J. Ward, and Z. Lu. NLM-Gene, a richly annotated gold standard dataset for gene entities that addresses ambiguity and multi-species gene recognition. *Journal of biomedical informatics*, 118:103779, 2021b. ISSN 1532-0480. doi: 10.1016/j.jbi.2021.103779.

R. Islamaj, R. Leaman, D. Cissel, C. Coss, J. Denicola, C. Fisher, R. Guzman, P. G. Kochar, N. Miliaras, Z. Punske, K. Sekiya, D. Trinh, D. Whitman, S. Schmidt, and Z. Lu. NLM-Chem-BC7: manually annotated full-text resources for chemical entity annotation and indexing in biomedical articles. *Database*, 2022, 12 2022. doi: 10.1093/database/baac102.

J. Johnson, M. Douze, and H. Jégou. Billion-scale similarity search with GPUs. *IEEE Transactions on Big Data*, 7(3):535–547, 2019. doi: 10.1109/TBDATA.2019.2921572.

D. Kartchner, J. Deng, S. Lohiya, T. Kopparthi, P. Bathala, D. Domingo-Fernández, and C. Mitchell. A comprehensive evaluation of biomedical entity linking models. In H. Bouamor, J. Pino, and K. Bali, editors, *Proceedings of the 2023 Conference on Empirical Methods in Natural Language Processing*, pages 14462–14478, Singapore, December 2023. Association for Computational Linguistics.

R. Leaman and Z. Lu. Taggerone: joint named entity recognition and normalization with semi-markov models. *Bioinformatics*, 32(18): 2839–2846, 2016. ISSN 1367-4803. doi: 10.1093/bioinformatics/btw343.

J. Li, Y. Sun, R. J. Johnson, D. Sciaky, C.-H. Wei, R. Leaman, A. P. Davis, C. J. Mattingly, T. C. Wiegers, and Z. Lu. BioCreative V CDR task corpus: a resource for chemical disease relation extraction. *Database*, 2016(baw068), 2016. ISSN 1758-0463. doi: 10.1093/database/baw068.

C. E. Lipscomb. Medical subject headings (mesh). *Bulletin of the Medical Library Association*, 88(3):265, 2000.

L. Luo, P.-T. Lai, C.-H. Wei, C. N. Arighi, and Z. Lu. Biored: a rich biomedical relation extraction dataset. *Briefings in Bioinformatics*, 23, 9 2022. doi: 10.1093/bib/bbac282.

L. Luo, C.-H. Wei, P.-T. Lai, R. Leaman, Q. Chen, and Z. Lu. Aioner: all-in-one scheme-based biomedical named entity recognition using deep learning. *Bioinformatics*, 39, 5 2023. doi: 10.1093/bioinformatics/btad310.

J. Luoma, K. Nastou, T. Ohta, H. Toivonen, E. Pafilis, L. J. Jensen, and S. Pyysalo. S1000: a better taxonomic name corpus for biomedical information extraction. *Bioinformatics*, 39, 6 2023. doi: 10.1093/bioinformatics/btad369.

A. Marzal and E. Vidal. Computation of normalized edit distance and applications. *IEEE Trans. Pattern Anal. Mach. Intell.*, 15: 926–932, 1993. URL https://api.semanticscholar.org/CorpusID:14851115.

S. Mohan and D. Li. MedMentions: A large biomedical corpus annotated with umls concepts. In *In Proceedings of the 2019 Conference on Automated Knowledge Base Construction (AKBC 2019)*, 2019. URL https://openreview.net/forum?id=SylxCx5pTQ.

E. Pafilis, S. P. Frankild, L. Fanini, S. Faulwetter, C. Pavloudi, A. Vasileiadou, C. Arvanitidis, and L. J. Jensen. The SPECIES and ORGANISMS resources for fast and accurate identification of taxonomic names in text. *PLOS ONE*, 8(6):e65390, 2013. ISSN 1932-6203. doi: 10.1371/journal.pone.0065390.

F. Scott. The NCBI Taxonomy database. *Nucleic Acids Research*, 40:D136–D143, 1 2012. ISSN 0305-1048. doi: 10.1093/nar/gkr1178.

M. Sung, H. Jeon, J. Lee, and J. Kang. Biomedical entity representations with synonym marginalization. In *Proceedings of the 58th Annual Meeting of the Association for Computational Linguistics*, pages 3641–3650, Online, July 2020. Association for Computational Linguistics. doi: 10.18653/v1/2020.acl-main.335.

C.-H. Wei and H.-Y. Kao. Cross-species gene normalization by pecies inference. *BMC Bioinformatics*, 12, 12 2011. doi: 10.1186/1471-2105-12-s8-s5.

C.-H. Wei, H.-Y. Kao, and Z. Lu. GNormPlus: An integrative approach for tagging genes, gene families, and protein domains. *BioMed Research International*, 2015:e918710, 2015. ISSN 2314-6133. doi: 10.1155/2015/918710.

C.-H. Wei, L. Luo, R. Islamaj, P.-T. Lai, and Z. Lu. GNorm2: an improved gene name recognition and normalization system. *Bioinformatics*, 39, 10 2023. doi: 10.1093/bioinformatics/btad599.

C.-H. Wei, A. Allot, P.-T. Lai, R. Leaman, S. Tian, L. Luo, Q. Jin, Z. Wang, Q. Chen, and Z. Lu. Pubtator 3.0: an ai-powered literature resource for unlocking biomedical knowledge. *Nucleic Acids Research*, 4 2024. doi: 10.1093/nar/gkae235.

H. Yuan, Z. Yuan, and S. Yu. Generative biomedical entity linking via knowledge base-guided pre-training and synonyms-aware fine-tuning. In *Proceedings of the 2022 Conference of the North American Chapter of the Association for Computational Linguistics: Human Language Technologies*, pages 4038–4048, Seattle, United States, July 2022. Association for Computational Linguistics. doi: 10.18653/v1/2022.naacl-main.296.
